# Supplementary material for: Patterns of Intron Gain and Loss in Fungi
Source: PLoS Biol. 2004 Nov 30;2(12):e422. doi: 10.1371/journal.pbio.0020422 (PMC532390; doi:10.1371/journal.pbio.0020422)
Supplement: Table S1 — Also available at http://genes.mit.edu/NielsenEtAl/. (4.3 MB ZIP). [file pbio.0020422.st001.zip › NielsenEtAl/html/420.html]

AN2938.1.NCU03280.1.MG00547.1.FG07060.1


```
 CLUSTAL W (1.82) Multiple Sequence Alignments - Introns Inserted


Sequence 1: NCU03280.1	445 aa
Sequence 2: MG00547.1	369 aa
Sequence 3: FG07060.1	372 aa
Sequence 4: AN2938.1	375 aa
Alignment Length: 513 aa
Number Identitical Residues: 119 aa
Alignment Score (without introns) 6327


MG00547.1 	------MATHTTDPELDSRP------------------SETTFTDGELNS--VIEATNE-
NCU03280.1	MSASNEVGAHTTGDFPQDREEFQTIAQSIQGVQDPEIVFPTSTRTGELPTEGREESNKE-
FG07060.1 	-------MIHDTGASINNEPS----------------KDPEPAESHSITDVEVVEALQKG
AN2938.1  	------------------------------------------------------------
          	                                                            

MG00547.1 	IISSTPAAVDLQIVED-VGSYYWDASTMGQLNKRS-~-----------------L0AAIA
NCU03280.1	DGESAKDQEEEKAIDS-EASHYWDASTMKPLSPASA0IIIFYENVQVTYHNSTLQ~AAIA
FG07060.1 	IREAASDNVREELINEGEASLYYDSLTMSPLNAMS-~-----------------A0AAIA
AN2938.1  	---------------------------MPRLSAAA-~-----------------Q0EAIN
          	                           *  *.  :                      ** 

MG00547.1 	RLRAYQSPPFPLWDRLPATRRAAVLVLLYADRAGELRVVITMRSATLRNFSGLKRTQDIH
NCU03280.1	RLRAFKPPPFPLWDRLPISRRAAVLLLLFADKRGDLRVVITMRAASMRSFS---------
FG07060.1 	RFRAYKPPSFPLWDRLPVRRRAAVLILLYADRHGDLRVVITMRAASLRNFS---------
AN2938.1  	RLRAFKPPPT-SYDLVPLSRRAAVLLLLYADAKGDLRVVLTIRASTLSSCMSLSS-----
          	*:**::.*.   :* :*  ******:**:**  *:****:*:*:::: .  . .      

MG00547.1 	LKGVFKLIWFELTIG~QAAFPGG~KADSVDESPY1QIARREAWEEIGLPMDDSKIPAPFV
NCU03280.1	--------------G~HAALPGG~KADSVEETPY1QIARREAWEEIGLPMDDSKLPSPFR
FG07060.1 	--------------G~HAAFPGG~KADDAQETPF1QIARREAWEEIGLPMDDSKLPKPFR
AN2938.1  	-------LIVTVRQR1QAALPGG1KSDSLDETPL~QTARREAHEEIGLPNLIQPLPPPFR
          	                :**:*** *:*. :*:*  * ***** ******   . :* ** 

MG00547.1 	IENLCYLPHSLARTGLVVRPCVAFLHPDPTKVDGSELPNVDETLIPRLDAKEVAAVFSAP
NCU03280.1	IEHLCYLPMNLARTELVVRPCVAFLHAD-DKVA----PAADESLIPRLNAKEVAAVFSAP
FG07060.1 	VEHLCYLPPSLARTHLVVRPCVAFLHAD-QRTAGEPAPTVEETMIPRLDAREVAAVFSAP
AN2938.1  	VEHLCEIPCSLARTELVVRPCVALLHTFDERTG--ENADPEITLIPRLDAREVAAVFTAP
          	:*:** :* .**** ********:**.   :.     .  : ::****:*:******:**

MG00547.1 	FHNFLKAQDEGTGP--------VPSGQWYEGRWTDYNDYRWRL~HYFYVPIDRQRVTRP-
NCU03280.1	FHNFLRAHDEVPKSDEDGSQEKPPPGKWYEGSWTNWHEERWRL~HFFYVPVNNQQVAKPG
FG07060.1 	FYNFLQANDLPPGPGE-----TLPEGQWYDGFWHSWKDHQWRV~HNFYVPVNNQSISKP-
AN2938.1  	FYDFLKLKPAGDEG-------------WYRGVWNEWWGTQWRM1HQFFVPVNPDKVVKPR
          	*::**: :                   ** * * .:   :**: * *:**:: : : :* 

MG00547.1 	--------KEREGGQAALAEPEES-----------------APEVRFKVWGMTGRMLVDA
NCU03280.1	SRKQLKAAEEKSSNQSAIINPEDDEDLPPPTA--ALARGDEEESERYKVWGMTARILVDA
FG07060.1 	---------RKDSEQSHLAEKLEKE---------------EEPDGRFKVWGMTARILVDA
AN2938.1  	P-----HHARQEEAVRDLEEQESKQQRSHQSQGQAAEQGRSDSVTRYRVFGMTARILVDA
          	.        .:.     : :  .... .  : ..:   . .    *::*:***.*:****

MG00547.1 	ARLAYGEEPEFEHNEDYGDEKMIN-------~---------------ELESQILET--KL
NCU03280.1	ATVAYGEEPEFEHNSHFGDERMIMGLDKLGM~LGE----------KTKKESVFTQE--DL
FG07060.1 	ARIAYAQEPGFEHNDNFGDEDIIAHADSLG-~---------------ELGEKVQEK--PH
AN2938.1  	ARIAYSTEPEFEHNRHSGDEELIARLRRRGR2HYLNTLRSVEVPICRPLISVYLQVRNSS
          	* :**. ** **** . *** :*      .     .:  : . .      .   :  .  

MG00547.1 	---------------~-------------------------
NCU03280.1	KKASEAAKKASEASK~M------------------------
FG07060.1 	EKKTEPG----DAAK~M------------------------
AN2938.1  	DNAAIPSEAKPNCAT0LVGLGLNLGLFSVAEIRLLGGSSAS
          	.. : ...  ...:.   . . . .  : :.    ..::::
```
